# Awareness of meaning and quest for meaning: The mechanisms between future orientation and prosociality among youth during pandemic

**DOI:** 10.3389/fpsyg.2022.1046803

**Published:** 2022-12-20

**Authors:** Wai-Kin Lui, Chi-Keung Chan, Kai-Hang Ng, Chi-Fai Raymond Chui, Nicolson Yat-Fan Siu, Chui-Shan Yung, Ka-Wing Lau

**Affiliations:** ^1^Department of Counselling and Psychology, Hong Kong Shue Yan University, Hong Kong, China; ^2^School of Arts and Humanities, Tung Wah College, Hong Kong, China; ^3^Department of Social Work, Hong Kong Shue Yan University, Hong Kong, China; ^4^Division of Social Science, School of Humanities and Social Science, The Hong Kong University of Science and Technology, Hong Kong, China

**Keywords:** meaning in life, presence of meaning, search for meaning, future orientation, prosocial tendency, Chinese youth

## Abstract

**Introduction:**

The positive relationship between future orientation and prosocial tendency has been consistently reported. However, the possible mechanism has not been examined yet. Previous research revealed the positive relationship between future orientation and meaning in life, as well as between meaning in life and prosocial tendency. Hence, it is hypothesized that the two components of meaning in life (presence of meaning and search for meaning) possibly mediate the relationship between future orientation and prosocial tendency.

**Methods:**

During the first half of 2020, 430 Hong Kong youths aged 15–35 (male: 30.5%; female: 69.5%) were recruited to participate an online cross-sectional survey. The survey included three scales: (1) The Scale for Measuring Adult’s Prosocialness, (2) Consideration of Future Consequence Scale, and (3) Meaning in Life Questionnaire.

**Results:**

The key findings showed that: (1) females had higher level of prosocial tendency than males, and (2) significant partial mediating effects of both presence of meaning and search for meaning on the relationship between future orientation and prosocial tendency. Nevertheless, the multi-group mediation model did not show significant gender difference.

**Discussion:**

These findings implied that future-oriented and meaning-focused interventions could possibly enhance youth’s sense of meaning in everyday life and foster their meaning searching tendency, which further strengthen the positive effect of their future orientation on prosocial tendency, even during life adversities.

## 1. Introduction

The total number of volunteers and the total time spent on volunteering showed a decrement trend globally. As compared to the time before COVID-19 pandemic, a lower volunteer rate in Australia was observed (Biddle and Gray, 2022), and the service time of United States volunteers decreased by 66% (Fidelity Charitable, 2020). During pandemic, more than a half of the non-government organizations have limited resources for operation (Sterling Volunteers, 2021). In Hong Kong, there was a sharp falling in the volunteer service hours in 2020 with a decrease of 13 million hours as compared to 2019 (Volunteer Movement, 2020). The decline of prosocial behaviors reflects the decreasing tendency to help people or fewer chances to perform prosocial acts due to limited operation of non-government organizations.

Prosocial behaviors other than helping behaviors are also particularly essential for a society, for instance, cooperation is a need for the survival of human beings. Prosocial behaviors work as a “social glue” that allows people to cooperate with harmony and productivity (Lay and Hoppmann, 2015). Furthermore, prosocial behaviors can support human beings to face global crises such as COVID-19 pandemic. For instance, employing protective measures is regarded as a form of prosocial behavior due to the other-orientated motivation to help reducing the infection and transmission of COVID-19 (Dinić and Bodroža, 2021).

Previous research has shown that future orientation is positively associated with prosociality (Strathman et al., 1994; Moore et al., 2001; Strobel et al., 2013; Li et al., 2019). Furthermore, meaning in life has been shown to be positively related with prosocial tendency (Lin, 2019; Liu and Zhang, 2020). Considering the rapid drop of prosocial acts globally during pandemic and the importance of cooperation to face life challenges, it is important to investigate the possible factors and mechanisms for enhancing prosociality when facing challenges and adversities in everyday life. It is not surprising that thinking about the future can contribute to prosocial tendency as a prosocial act may involve the trade-off between limited short-term benefits and greater long-term benefits (Romer et al., 2010). However, although the long-term benefits of a prosocial act can be greater, these benefits may not be meaningful to an individual. Therefore, it is possible that, after thinking about the consequences of a particular prosocial act, the individual evaluates whether the outcomes are meaningful or not according to his/her meaning system to decide whether to perform the prosocial act. And that is a meaning making process that assigns meaning to the events and stimuli (Märtsin, 2019). Also, meaning in life involves the integration and comprehension of the anticipated future to construct meaning in life. Nevertheless, the possible mediation effect of meaning in life in the relationship between future orientation and prosociality has not been examined yet. Hence, an investigation of this proposed mediation model can provide some recommendations for enhancing prosociality of young adults. Hence, the main purpose of this study is to examine whether the two components of meaning in life, presence of meaning and search for meaning, can significantly mediate the relationship between future orientation and prosocial tendency.

## 2. Literature review

### 2.1. Prosocial tendency

Prosocial acts refer to human behaviors that eliminate suffering and promote well-being of others, and prosocial tendency reflects how likely an individual would perform a prosocial act (Eisenberg and Miller, 1987; Caprara et al., 2005). There is a variety of prosocial behaviors, like offering help to others, benevolent actions, and collaboration with others. Interestingly, recent research (Dinić and Bodroža, 2021) argued that adopting protective measures against COVID-19 transmission should also be regarded as a type of prosocial behavior during the pandemic since the motivation behind these acts is to protect others from infection.

Furthermore, Eisenberg et al. (2002) have demonstrated that prosocial behaviors were crucial for increasing social bonding and relatedness, maintaining social harmony, and supporting the development of the individuals and the society. Prosociality is also highly related to psychological wellbeing, happiness, and life satisfaction (Lauri and Calleja, 2019). Furthermore, prosociality can be one of the protective factors against negative psychological outcomes like depression and anxiety (Raposa et al., 2016; Taylor et al., 2017) and negative behavioral outcomes like substance abuse (Carlo et al., 2011). Therefore, prosocial tendency appears to be an influential factor for mental wellness.

### 2.2. Future orientation and prosocial tendency

Future orientation describes how often and how likely an individual thinks about and adjusts oneself for the future (Nurmi, 2005; Seginer, 2009). Particularly, individuals with higher level of future orientation are more likely to consider future, anticipate future outcomes, and have a plan before any actions; whereas those with lower level of future orientation are more likely to focus on the present and immediate consequences and do not want to think or plan ahead for the future (Nurmi, 2005; Seginer, 2009). Importantly, future orientation is associated with emotional, attitudinal, cognitive, behavioral aspects of an individual, and involves the process of decision making (Nurmi, 2005). Therefore, future orientation is closely linked to people’s optimistic or pessimistic thoughts in their daily lives. Various terms like future time orientation, possible future self, future time perspective, and consideration of future consequences have been used interchangeably to conceptualize future orientation (Seginer, 2009).

Empirical research showed that future orientation is associated with prosociality. Moore et al. (2001) find that thinking about possible future manifested in delayed gratification which supports one’s prosocial behaviors, for instance, sacrificing one’s current needs to benefit others’ needs. Furthermore, future orientation has been found to be a key predictor of prosociality (Strathman et al., 1994; Strobel et al., 2013), the mediators on the relationship between awe and prosocial behavior (Li et al., 2019) and the mediator between reputational motivation and prosocial behavior (Choi, 2020). Furthermore, higher level of future orientation helps individuals to resist the temptation to have immediate rewards for a greater reward in the future (Romer et al., 2010). Nevertheless, future-based cost-effect analysis is not sufficient to explain all prosocial behaviors; for instance, devoting oneself to the propagation of religion can provide others with mental comfort and inner peace, but the missionary does not receive any substantial rewards. One important question should be stressed here is what make people to have such costly prosociality. Given that religion is one of the sources constructing meaning in life (Fletcher, 2004), meaning in life is a possible reason fostering people to do these prosocial acts.

### 2.3. Meaning in life: The presence of and the search for meaning

Meaning in life is a subjective feeling and experience of the sense of significance and worthiness toward oneself, the life, and the world, and involves sensemaking to interpret and organize stimulations and personal experiences (Steger, 2009). Three constructs of meaning in life are proposed – comprehension, purpose, and significance (Steger, 2009; George and Park, 2016; King and Hicks, 2021). First, comprehension refers to the consistency of how an individual interprets, comprehends, and integrates, his/her experiences, the self, and the world. Second, purpose refers to the perception that an individual is moving toward to valued goals. Third, significance refers to the belief that an individual is important, and influential to others and the world.

Furthermore, Steger et al. (2006) suggested that presence of meaning and search for meaning reflected the two dimensions of meaning in life. Presence of meaning in life demonstrates the perceived meaningfulness, worthiness, and importance. On the other hand, search for meaning in life reflects the tendency to seek, develop, and enhance the understanding and the sense of meaning in life. Steger et al. (2006) suggested that the presence of meaning and search for meaning are not mutually exclusive. That is, people who have higher level of the presence of meaning can have either high or low level on the search for meaning, and vice versa.

Chu and Fung (2021) proposed two types of meaning searching process: growth searching and deficiency searching. Growth searching is to deepen the understanding and look for further meaning with well-established sense of meaning, whereas deficiency searching is to reduce the anxiety resulted from the lack of the sense of meaning. Distress may be the short-term effect of deficiency searching, especially during life adversities when there is a lack of the awareness of meaning. In the long-run, people who adopt deficiency searching are more likely to experience the sense of meaning as compared to those who have lower motivation to search for meaning (Chu and Fung, 2021). The two types of searching process can also be considered as a continuous meaning making process. That is, people with low level of presence of meaning usually experience deficiency searching and establish sense of meaning in the long run; then, once an individual establishes a sense of meaning, growth searching process is adopted for further development of meaning or searching for other meaning. Most importantly, this continuous and dynamic searching process of meaning in life (Brown, 2000; Seligman, 2004; Chu and Fung, 2021) can further develop and deepen meaning from focusing on oneself to others, supporting and sustaining the belief of the significance of one’s existence (Steger, 2012; King and Hicks, 2021).

### 2.4. Meaning in life and future orientation

Research revealed that future orientation was associated with the presence of meaning in life (Hicks et al., 2012; Baumeister et al., 2020; Miao et al., 2021). Theoretically, the constructs of meaning in life are relevant to future orientation. “Comprehension” involves the integration of the past, present, and the anticipated future, and “purpose” involves goals setting and plans that help to achieve goals; the belief that one’s long-lasting influence (i.e., “significance”) is also related to future anticipation (Steger, 2009; George and Park, 2016; King and Hicks, 2021). Thus, future-orientated individuals tend to have higher sense of meaning due to the increased sense of comprehension, purpose, and significance derived from thinking about future, anticipating the future, and planning for the future.

Moreover, future orientation also strengthens meaning searching tendency. Leshkovska and Shterjovska (2014) argued that future time perspective contributed to the tendency to search for further or deeper meaning in life. Also, youths are more likely to search for meaning from the future goals to develop a sense of purpose and significance to overcome the rumination of negative past experiences (Leshkovska and Shterjovska, 2014).

### 2.5. Meaning in life and prosocial tendency

Studies (Van Tongeren et al., 2016; Klein, 2017) revealed that prosocial acts fostered the presence of meaning by increasing the sense of self-worth, self-transcendence, and relationship satisfaction. Nevertheless, more studies proposed a reversed relationship between the presence of meaning and prosocial tendency. Previous research found that meaning could significantly enhance youths’ volunteering beliefs, tendencies, and behaviors (Law and Shek, 2009) and boosted adolescents’ altruism (Shek et al., 1994). Furthermore, meaning in life can promote civic engagement *via* participating in voluntary organizations and increase sense of social connectedness (Routledge and FioRito, 2021). As aforementioned, meaning in life *per se* involves self-transcendent property which highlight the perceived significance and influence of oneself to the others and the world (Steger, 2009; George and Park, 2016; King and Hicks, 2021). Researchers (Brown, 2000; Seligman, 2004) also suggested that meaning in life involved the contribution to people or things other than oneself. Therefore, it is not surprising that presence of meaning positively predict prosocial tendency.

The positive association between meaning in life and prosocial tendency indicates that people searching for meaning would engage in prosocial acts to experience meaningfulness. Particularly, meaning-seekers show the strong intention to engage in civic activities (Lin, 2019) because these activities can enhance their sense of meaning through increasing social connectedness (Ohmer, 2007), self-esteem (Brown et al., 2012), and sense of competence in organizing their lives (Zimmerman and Rappaport, 1988), which are highly associated with meaning in life (Heine et al., 2006). In addition, meaning-seekers engage in high-cost prosocial acts like kidney donation (Dakin et al., 2021) probably because these prosocial behaviors with higher costs are viewed as having more valuable and significant meaning (Olivola and Shafir, 2011; Inzlicht et al., 2018).

### 2.6. Future orientation, meaning in life, and prosocial tendency

Ellis (1991) ABC model has been widely used for the conceptualization of psychopathology, which highlights the irrational belief regarding events on contributing maladaptive behavioral and mental consequences (Bernard et al., 2010). Nevertheless, the model not only provides the conceptualization for problem behaviors and mental illness, but also motivates adaptive behaviors (e.g., Malkinson, 2010), and even wellness (Bernard et al., 2010). The model consists of three components - activating event (A-component), belief (B-component), and consequences (C-component). The A-component stands for any external and internal activating events, and the B-component refers to the interpretations and understandings of activating events; further, based on the interpretation and understandings made, an individual is likely to act or to react as C-component (Ellis, 1991).

**Figure 1:**
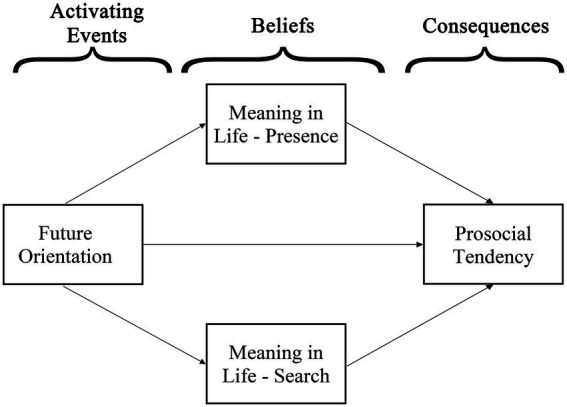
A hypothetical model for the relationships among major variables based on the ABC model (Ellis, 1991).

The present study suggests that the relationships among future orientation, meaning in life, and prosocial tendency can be conceptualized with Ellis (1991) ABC model (see Figure 1). Considering future orientation provides rich internal stimulations (e.g., goal seeking, anticipated future, and possible self), future orientation can be considered as the source of A-component. Furthermore, meaning in life can be considered as the B-component. Belief is defined as the subjective feelings and cognition regarding oneself and the environment around (Ajzen, 1995), which is highly similar to the construct of meaning in life defined by Martela and Steger (2016). Comprehension is about making sense of and having a sense of predictability and recognizability toward oneself, one’s life, and the world, whereas purpose and significance are about the value judgements on what is good, and valuable (Martela and Steger, 2016). It is worth noting that the sense of comprehension, and value judgements are belief, which represents unique and subjective understanding and interpretations for everything. Additionally, to decide whether the future outcomes are meaningful and valuable, one likely refers to his/her meaning system to make such judgements (Märtsin, 2019). Hence, given meaning in life by itself as a set of beliefs, and the process of meaning making, it is plausible that meaning in life is the B-component. On the other hand, the tendency to search for meaning can be motivated by anticipating future (Leshkovska and Shterjovska, 2014). Therefore, it is likely that both the presence of meaning and search for meaning are the B-component. Furthermore, considering the self-transcendent nature of meaning in life (Brown, 2000; Seligman, 2004), and the meaning-enhancing effect of prosociality (Zimmerman and Rappaport, 1988; Ohmer, 2007; Brown et al., 2012; Lin, 2019), those with higher levels of the presence of meaning and/or search for meaning, are more likely to have higher prosocial tendency. Alternatively, when the possible outcomes of a particular prosocial act are judged to be valuable and meaningful, an individual is more likely to have a higher level of prosocial tendency to perform the act. And the increased prosocial tendency and prosocial acts are the C-component.

### 2.7. The present study

The present study aims to investigate the relationships among future orientation, meaning in life, and prosocial tendency. Future orientation, as the activating events, can increase the source to comprehend one’s living, set life direction, and establish the belief of one’s importance, so as to construct a sense of meaning. Furthermore, the presence of meaning, as the belief, fosters prosocial tendency due to the self-transcendence nature of meaning in life. Meanwhile, future orientation can contribute to one’s meaning-searching tendency to seek greater and bigger meaning and gain deeper understanding of meaning in life. Consequently, due to this urge for a sense of meaning, a meaning-seeker is more likely to have high prosocial tendency. Thus, the increased prosocial tendency is the consequence of the presence of meaning and search for meaning generated by future orientation. Notably, the presence of meaning contributes to prosocial tendency due to the other-orientated meaning, whereas the search for meaning contributes to prosociality due to the need for establishing greater meaning. Hence, presence of meaning and search for meaning can be considered as two independent mediating processes. Therefore, the hypotheses of the present study include: (1) future orientation has significant positive relationship with prosocial tendency, (2) presence of meaning significantly mediates the relationship between future orientation and prosocial tendency, and (3) search for meaning significantly mediates the relationship between future orientation and prosocial tendency.

## 3. Materials and methods

### 3.1. Participants

An online survey was administrated from February to June 2020 during the first wave of COVID-19 pandemic in Hong Kong. Through snowball and convenient sampling, 465 respondents were initially recruited, but then 14 participants were excluded because they did not complete the survey and 17 participants were excluded because the time duration for completing the survey was less than 180 s (3 min). Furthermore, two underaged (<15 years old) and two overaged respondents (>30 years old) were also excluded. Therefore, the final valid data contained 430 Hong Kong youth participants (see Table 1 for major characteristics) with an age range from 15 to 30 years old (*M* = 22.96, *SD* = 3.19). The age distribution was 11.4% for 15–19 years old, 60.5% for 20–24 years old, and 28.1% for 25–30 years old. Approximately 30.5% of the participants were males (*n* = 131) and 69.5% were females (*n* = 299). Most of the participants hold a bachelor’s degree (*n* = 375, 87.5%), and were students (*n* = 224, 52.1%). According to the 2021 population census report conducted by the Census and Statistics Department, The Government of the Hong Kong Special Administrative Region Census and Statistics Department (2022), the total number of youth population (aged 15–30) was 1,149,503. The age distribution was 23.0% for 15–19 years old, 28.4% for 20–24 years old, and 48.6% for 25–30 years old. 51.4% of the population aged 15–30 were female. The group of 20–24 years old was over-sampled in this sample because more emerging adults were recruited in the present study.

**Table 1:** Major characteristics of participants.

Demographic Variables	*n*	*%*	*M* (*SD*)	Range
**Gender**				
Male	131	30.5		
Female	299	69.5		
Age			22.96 (3.19)	15–30
**Education**				
Secondary 1–3	5	1.2		
Secondary 4–6	22	5.1		
Bachelor	375	87.2		
Master or above	28	6.5		
**Job status**				
Student	224	52.1		
Full-time job	158	36.7		
Part-time job	18	4.2		
Self-employed	3	0.7		
Unemployed	26	6.0		
Other	1	0.2		

### 3.2. Measures

The online survey consisted of four parts including demographics, the Consideration of Future Consequence Scale (Joireman et al., 2012), the Meaning in Life Questionnaire (Steger et al., 2006), and the Scale for Measuring Adult’s Prosocialness (Caprara et al., 2005). The online survey was conducted with Chinese versions. The required and collected demographic data included age, gender, education level, and job/study status.

The consideration of future consequences subscale of Consideration of Future Scale (Joireman et al., 2012) was used to measure the degree to which an individual think about the future consequences. The scale consists of five items and the respondents answered each item on a 7-point scale – from 1 (Extremely Uncharacteristic) to 7 (Extremely Characteristic). The reliability of the scale in the present study was.77.

The Meaning in Life Questionnaire (Steger et al., 2006) was adopted to measure the two dimensions of the meaning in life, that is, presence of meaning and search for meaning. The presence of meaning indicates the extent to which an individual experience or feels his/her life is worthy, meaningful, and significant, whereas the search for meaning indicates the tendency to search for meaning in life. Both the presence of and the search for meaning subscales consist of five items respectively, and the respondents rated from 1 (Absolutely Untrue) to 7 (Absolutely True). The reliability of the presence subscale in the present study was 0.88, and that of the search subscale was.88.

The Scale for Measuring Adult’s Prosocialness (Caprara et al., 2005) was used to measure the prosocial tendency of an individual. It is worth noting that the scale measures prosocial tendency, but not actual prosocial behaviors. The scale includes 16 items rated from 1 (Never) to 5 (Always). The reliability of the scale in the present study was 0.92.

### 3.3. Procedures

The link to the online survey was sent *via* email and social media messages to universities students and the public, and the receivers were requested to share the link if possible (snowball sampling). Prior to filling the survey, each respondent was required to read the informed consent form and indicate their consent to participate in the study. After obtaining the consent form, each participant was required to complete the online questionnaire with the measures described above. The collected data were analyzed using SPSS 26 and AMOS 22 statistical packages. The correlation analysis of the key variables was conducted to examine the bivariate relationships among future orientation, presence of meaning, search for meaning, and prosocial tendency. Then, a path analysis was employed to test the hypothetical model with two mediators (presence of meaning and search for meaning) on the relationship between future orientation and prosocial tendency, respectively. Finally, multiple-group path modeling analysis was conducted to investigate whether there are differential path estimates between males and females for the two mediators.

## 4. Results

### 4.1. Descriptive statistics

Tables 1, 2 show the descriptive statistics of all measures for the final sample, and Table 2 shows the results of the independent sample t-tests on major measures between males and females. The prosocial tendency of females (*M* = 59.78, *SD* = 8.55) was significantly greater than males (*M* = 57.58, *SD* = 10.29), *t* (212.17) = −2.14, *p* = 0.03. However, no significant gender differences were found on future orientation (*t* (428) = 0.42, *p* = 0.67), presence of meaning (*t* (214.25) = −0.48, *p* = 0.63), and search for meaning (*t* (428) = −1.03, *p* = 0.30).

**Table 2:** Demographics and *t*-test for possible gender differences in major variables.

Variable	All subjects	Male	Female	*t*	*df*	95% CI	
	*M* (*SD*)	*M* (*SD*)	*M* (*SD*)			LLCI	ULCI
Age	22.96 (3.19)	23.07 (3.49)	22.92 (3.05)	0.455	428	−0.505	0.810
PT	59.11 (9.16)	57.58 (10.29)	59.78 (8.55)	2.140*	212.174	−4.218	−0.173
FO	23.99 (4.52)	24.13 (4.31)	23.93 (4.62)	0.422	428	−0.732	1.132
MILP	23.21 (6.05)	22.98 (6.78)	23.31 (5.71)	−0.476	214.254	−1.660	1.014
MILS	26.20 (4.95)	25.83 (5.25)	26.37 (4.82)	−1.033	428	−1.555	0.484
Group size	*N* = 430	*N* = 131	*N* = 299				

PT, prosocial tendency; FO, future orientation; MILP, the presence of meaning in life; MILS, the search for meaning in life. *p < 0.05; **p < 0.01; ***p < 0.001.

Further, the correlations among the measures for the entire sample is presented in Table 3. Future orientation was significantly and positively correlated with prosocial tendency (*r* = 0.341, *p* < 0.001), presence of meaning (*r* = 0.326, *p* < 0.001), and search for meaning (*r* = 0.303, *p* < 0.001). Also, prosocial tendency was positively correlated with presence of meaning (*r* = 0.329, *p* < 0.001), and search for meaning (*r* = 0.318, *p* < 0.001). Furthermore, presence of meaning was positively correlated with search for meaning (*r* = 0.170, *p* < 0.001).

**Table 3:** Pearson’s correlation coefficient of the questionnaire measures.

Variable	FO	MILP	MILS	PT
FO	-			
MILP	0.326***	-		
MILS	0.303***	0.170***	-	
PT	0.341***	0.329***	0.318***	-

*N* = 430; PT, prosocial tendency; FO, future orientation; MILP, the presence of meaning in life; MILS, the search for meaning in life. *p < 0.05; **p < 0.01; ***p < 0.001.

### 4.2. Path analysis

**Figure 2:**
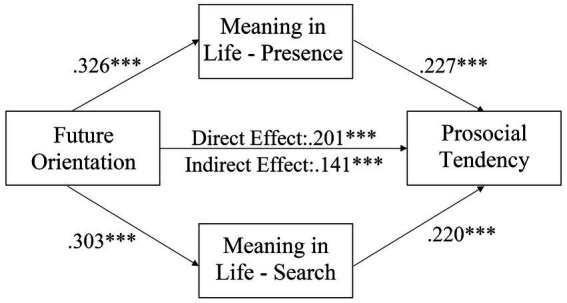
A hypothetical model for the relationships among major variables. *p < 0.05; **p < 0.01, ***p < 0.001.

**Figure 3:**
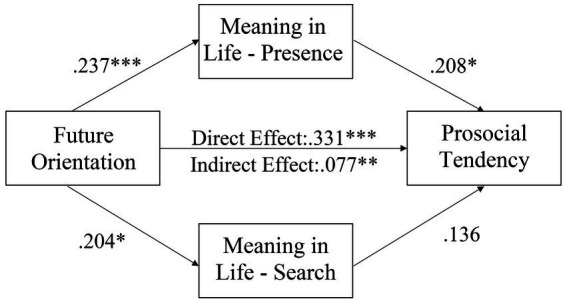
A hypothetical model for the relationships among major variables (male sample). *p < 0.05; **p < 0.01, ***p < 0.001.

**Figure 4:**
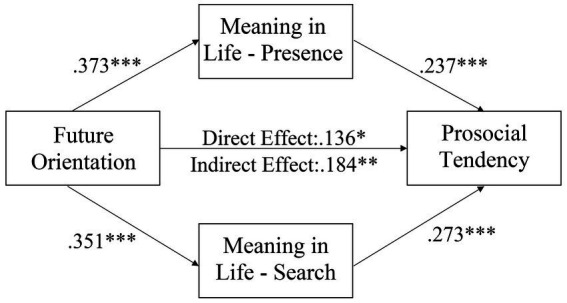
A hypothetical model for the relationships among major variables (female sample). *p < 0.05; **p < 0.01, ***p < 0.001.

Figure 2 shows the results of path analysis of the hypothesized model for the full sample. The hypothetical path model had very good-fit, *χ*^2^ (1, *N* = 430) = 2.673, *p* = 0.102; *χ*^2^/*df* = 2.673; GFI = 0.997; AGFI = 0.969; TLI = 0.947; CFI = 0.991; RMSEA = 0.062, with 95% C.I [0.000, 0.158]. The direct effect of future orientation on prosocial tendency was significant (*β* = 0.141, 95% CI [0.098, 0.190], *p* < 0.001). Then, future orientation significantly predicted presence of meaning (*β* = 0.326, 95% CI [0.238, 0.408], *p* < 0.001) and search for meaning (*β* = 0.303, 95% CI [0.212, 0.386], *p* < 0.001) and prosocial tendency (*β* = 0.201, 95% CI [0.112, 0.292], *p* < 0.001); presence of meaning (*β* = 0.227, 95% CI [0.140, 0.313], *p* < 0.001) and search for meaning (*β* = 0.220, 95% CI [0.135, 0.306], *p* < 0.001) significantly predicted prosocial tendency. The mediation model is significant (*β* = 0.141, bias-corrected and accelerated confidence interval [0.098, 0.190], *p* < 0.001). Concerning the significant direct effect and indirect effect of future orientation on prosocial tendency, the dimensions of meaning in life partially mediate the relationship. We further conducted a multi-group analysis to test any gender differences for the parameter estimates on the hypothesized model. Figures 3, 4 show the hypothetical mediation models for male and female sample, respectively. Results showed that there was no gender difference for the multi-group mediation model (Δ*χ*^2^(*df* = 5) = 7.786, *p* = 0.168).

## 5. Discussion

This study investigates the mediation roles of presence of meaning and search for meaning in the relationship between future orientation and prosocial tendency with Hong Kong youth during the first wave of pandemic. The findings of this study supported all the hypotheses, and no gender difference was found. The results of path analysis indicated the significant partial mediation effects of presence of meaning and search for meaning on the relationship between future orientation and prosocial tendency. Particularly, these results indicated that people with higher level of future orientation are more likely to have higher level of presence of meaning and/or search for meaning and, further, have higher level of prosocial tendency. In other words, the positive relationship of future orientation with prosocial tendency are partially explained by the presence of meaning and the search for meaning. These findings are consistent with the previous studies regarding the positive associations between future orientation and presence of meaning (Hicks et al., 2012; Baumeister et al., 2020; Miao et al., 2021), and search for meaning (Leshkovska and Shterjovska, 2014) as well as the positive associations between prosocial tendency and presence of meaning (Van Tongeren et al., 2016; Klein, 2017; Liu and Zhang, 2020), and search for meaning (Zimmerman and Rappaport, 1988; Ohmer, 2007; Brown et al., 2012; Lin, 2019; Dakin et al., 2021).

Theoretically, ABC model (Ellis, 1991) is proposed in this study for interpreting the mediation model of meaning in life on the relationship between future orientation and prosocial tendency. For the activating events, young people with higher future orientation tend to anticipate future and imagine possible self, which are the internal stimulations that act as the source for the belief on meaning-making. Those future anticipations and imaginations motivate Chinese youth to experience a sense of meaning and construct a belief regarding one’s meaning or the reflection of one’s meaning system. One can feel the sense of meaning because of the internal stimulations, the level of presence of meaning would be further enhanced. Alternatively, it is also possible that the youth refer to their meaning to decide whether the possible future outcomes are meaningful and valuable, which is a meaning making process (Märtsin, 2019). On the other hand, if one lacks the sense of meaning in the present life, the future simulations could contribute to the meaning searching motivation and tendency, from deficiency search to growth search in meaning through a self-transcendent process (Chu and Fung, 2021). Also, search for meaning include the belief of the existence of meaning rather than meaninglessness. As a result, future orientation of youth can further enhance their prosocial tendency through cultivating their awareness of the presence of meaning and the urge to search for greater and bigger meaning through a self-transcendent process and reframe a sense of meaning in their life.

Although the results also found females were more likely to have higher level of prosocial tendency than males which is consistent with the previous studies (e.g., Beutel and Marini, 1995; Beutel and Johnson, 2004; Carlo et al., 2012), the multiple-group path modeling analysis did not find any gender differences for the hypothetical mediation model. Also, the present study did not find any gender differences on future orientation (e.g., Greene and Wheatley, 1992; Mello and Worrell, 2006; Borowsky et al., 2009) and the two components of meaning in life (e.g., Yu et al., 2017; Dhanjal, 2019; Xi et al., 2022) proposed found in previous studies.

A possible explanation is the raised awareness and improvement of gender equality in Chinese societies. Several previous studies suggested that gender differences on future orientation (Seginer, 1988; Nurmi et al., 1995; Seginer and Halabi-Kheir, 1998) and meaning in life (Grouden and Jose, 2014) could be explained by gender inequality and cultural socialization. The insignificant gender differences on future orientation, meaning in life, and the hypothetical model, suggesting that both male and female youth in Hong Kong are raised in a society with relatively better gender equality. However, females show higher prosocial tendency than males in this study, and that can possibly be explained by the neurological differences. Particularly, females were found to have higher level of inter-hemispheric and modular connection than males (Ingalhalikar et al., 2014), so that the region related to social cognition can be activated more easily due to the connectivity. Hence, females are more likely to have higher level of prosocial tendency than males (Glimcher and Fehr, 2013). However, future neuropsychological studies should be conducted to futher investigate this possible explanation.

The ABC model (Ellis, 1991) on the relationships among future orientation, meaning in life, and prosocial tendency provides a framework to understand how one’s anticipation of future and interpretation the meaning of the daily life events can influence his/her prosocial attitudes and behaviors. The anticipated future or imagined possible self are the daily life stimulators for the youths. Even youths with low level of future orientation, and facing life adversities (e.g., pandemic) would think about the future. Hence, the future in mental representation *per se* is the daily source of the presence of meaning, and the fuel to the meaning searching motivation, and this iterative awareness of meaning, meaning-searching, and meaning-making process in everyday life can further enhance the prosocial tendency. In other words, the enhancement on presence of meaning and search for meaning can explain and strengthen the effect of future orientation on prosocial tendency.

## 6. Implications

The findings of the present study implied that promoting and enhancing the meaningful life of youth through thinking about their future can help them to cultivate their sense of comprehension, significance, and purpose in their daily lives, and motivate them to seek for greater meaning in the process, which further strengthen their tendency to engage in prosocial acts. For example, requesting youth to write their best possible selves during life difficulties and adversities may help to enhance their future orientation (King, 2001). Most importantly, the best possible selves reflect the youths’ meaning in life and value as the imagined future consists of the achievements of life goals (King, 2001). Also, meaning centered interventions provide reflective space for youth to freely explore their meaning in life (Lim and Kang, 2018). Therefore, by highlighting the meaning reflected in the best possible selves, youth can increase their sense and awareness of meaning in their daily life and be motivated to search for greater or other-oriented meaning, which in turn further increase their prosocial tendency. Furthermore, confronting daily crises like financial difficulties, relational dissolutions, or global crises like pandemic, youth can explore meaning of these adversities and develop their meaning from anticipating future, which can contribute to their prosocial tendency to help the needy to overcome the diversities and promote growth.

## 7. Limitations and future direction

The present study has two major limitations. First, the recruited participants were mainly youths in Hong Kong, so the findings and implications of the study cannot be generalized to the youth in other Chinese societies. Furthermore, given the cross-sectional nature of this study, no causal relationships among future orientation, meaning in life, and prosocial tendency could be claimed. Therefore, a longitudinal study with meaning-focused future-orientation intervention to enhance youth’s prosociality must be rigorously designed and validated to testify the implications of the current study.

In conclusion, findings of the present study support that the mediating roles of presence of meaning and search of meaning on the relationship between future orientation and prosocial tendency. In other words, through meaning-focused future-oriented interventions, it is possible to enhance youth to experience their daily life as meaningful and further search for deeper and greater meaning in their present lives. Also, even facing life adversities and difficulties, enhancing the sense of meaningfulness and promoting meaning-seeking tendency by thinking and anticipating future, can increase youth’s prosocial tendency and actions to contribute to the harmony and development of family, community, and society to face any life and global crises and challenges facing in our everyday life.

## Data availability statement

The raw data supporting the conclusions of this article will be made available by the authors, without undue reservation.

## Ethics statement

The studies involving human participants were reviewed and approved by Human Research Ethics Committee of the Hong Kong Shue Yan University. Written informed consent to participate in this study was provided by the participants’ legal guardian/next of kin.

## Author contributions

W-KL contributed to study conceptualization, results interpretation, data analysis and writing the original manuscript. C-KC contributed to survey design, administration and overseeing of the research, revision and editing of the manuscript. K-HN contributed to data analysis. C-FRC contributed to survey design. NS contributed to scale validation. C-SY contributed to revising and editing the manuscript, and checking of citations and references. K-WL contributed to checking of citations and references, and generation of tables and figures. All authors contributed to the article and approved the submitted version.

## Conflict of interest

The authors declare that the research was conducted in the absence of any commercial or financial relationships that could be construed as a potential conflict of interest.

## Publisher’s note

All claims expressed in this article are solely those of the authors and do not necessarily represent those of their affiliated organizations, or those of the publisher, the editors and the reviewers. Any product that may be evaluated in this article, or claim that may be made by its manufacturer, is not guaranteed or endorsed by the publisher.
